# Cascading effects of seed-stem-individual spatial patterns along a grazing gradient

**DOI:** 10.3389/fpls.2023.1137726

**Published:** 2023-03-15

**Authors:** Zhou Qin-Yuan, Dong Quan-Min, Wang Fang-Cao, Liu Yu-Zhen, Feng Bin, Yang Xiao-Xia, Yu Yang, Zhang Chun-Ping, Cao Quan, Liu Wen-ting

**Affiliations:** Qinghai Provincial Key Laboratory of Adaptive Management on Alpine Grassland, Academy of Animal Science and Veterinary Medicine, Qinghai University, Qinghai Academy of Animal Science and Veterinary Medicine, Xining, China

**Keywords:** seed traits, spatial pattern, kobresia humilis, grazing, stem resource allocation

## Abstract

Studying the seed trait–stem trait–individual spatial pattern system is helpful for understanding the developmental direction of plant dynamics and populations under grazing disturbance as well as the antagonistic relationship between animals and plants, but few systematic analyses of this spatial pattern system have been carried out. *Kobresia humilis* is the dominant species in alpine grasslands. We studied *K. humilis* seed traits and their relationship with *K. humilis* reproductive individuals, the relationship between reproductive and vegetative stems, and the weights and spatial patterns of reproductive and nonreproductive individuals under four grazing treatments: no grazing (control), light grazing, moderate grazing and heavy grazing. We explored the relationship among seed size and seed number with reproductive stems and vegetative stems along the grazing gradient and assessed the spatial pattern changes between reproductive and nonreproductive individuals. The results showed the following: (1) Seed size increased with increasing grazing intensity, and the coefficient of variation for seed size and seed number in the heavy grazing treatment was greater than 0.6. (2) The structural equation model showed that grazing treatment had a positive effect on seed number, seed size and reproductive stem number and a negative effect on reproductive stem weight. (3) Grazing treatment did not affect the resource allocation to reproductive stems and vegetative stems per unit length of reproductive *K. humilis* individuals. (4) Compared with the number of reproductive individuals in the no grazing treatment, the number in the heavy grazing treatment decreased significantly, and the negative correlation between reproductive individuals and nonreproductive individuals changed from a full-scale negative correlation to a small-scale negative correlation and a large-scale positive correlation. Our study showed that grazing could activate and change the resource allocation pattern of dominant species in a grassland and have significant positive effects on reproductive stem number, reproductive stem weight, seed number and seed size. Along a grazing intensity gradient, with the increase in distance between reproductive and nonreproductive individuals, the transformation of intraspecific relationships from a negative correlation to a positive correlation is an ecological strategy conducive to population survival.

## 1 Introduction

Disturbance has been defined as an important mechanism that promotes the maintenance of plant community species diversity (Connell, 1978; Kadmon and Benjamini, 2006). In grassland ecosystems, grazing disturbance is the main process that affects plants, pasture utilization and natural conservation and is essential for grassland maintenance, productivity, economic utilization and biodiversity (Asner et al., 2004; Lezama et al., 2014). A large number of studies have reported that the top-down control of plant tissues by large herbivores leads to ecological conditions and processes such as an imbalance between plant resource demand and production (Orr et al., 2020), a redistribution of nutrients (Zhang et al., 2020), and a reduction in carbon assimilation, thereby shaping many cascading effects, including those related to individual reproduction, survival, and spatial patterns of dominant species (Liu et al., 2020).

Plant functional traits are closely related to plant environmental adaptability and ecosystem structure and function (Kraft et al., 2015). Environmental changes affect the expression of plant functional traits, and plant functional traits affect the function of ecosystems. The shaping of plant roots, stems and leaves by the environment changes the morphological, physiological and quantitative characteristics of plant organs so that plants can better adapt to environmental changes. Therefore, changes in plant functional traits have indicative and predictive effects on environmental changes (Li et al., 2021). There are many views on the responses of plant functional properties to grazing. Liu et al. (2018) suggested that grazing livestock, through the direct effect of feeding, make plants shorter, leaves shorter and narrower, internodes shorter, stems and leaves stiffer, and shrub crowns smaller, thus reducing the number and size of dominant plant seeds in a grassland and significantly increasing the size variation among seeds of the same individual. Some studies also suggest that grazing improves the ability of plants to allocate resources to reproductive stems during the reproductive period (Liu et al., 2020), thereby maintaining their efficiency for sexual reproduction. Because seeds will eventually be dispersed through the digestive tracts of animals, herbivores do not affect the number of seeds of plants and even increase the success rate of seed germination and diffusion distance (Mackay et al., 2021). The study of plant functional traits can provide an in-depth understanding of the reproductive strategies and dispersal mechanisms of plants that are driven by biotic and abiotic factors (Funk et al., 2017) as well as of the construction patterns of plant communities (Wang et al., 2021).

Species spatial patterns have always been a foundation of ecological research (Watt, 1947; Wiegand et al., 2007; Velázquez et al., 2016). Studies have shown that the competition of plants for limited resources is the main factor driving the coexistence and dynamic changes in species such as herbs and shrubs. The inhibition and exclusion (intraspecific competition) of different individuals in the same population have an important impact on the spatial distribution of plant individuals (Erfanifard et al., 2018). There are two views on the effect of simultaneous grazing at the individual scale. One view is that the density and/or characteristics of adjacent plants can directly change the resistance and tolerance levels of livestock focal plants through competition or through joint resistance or sensitivity (Agrawal and Fishbein, 2006). Another consideration is that spatial associations with neighbouring plants can reduce the likelihood of damage and increase the apparent resistance of focal plants if neighbours reduce their apparent resistance to herbivores or if herbivores prefer to eat more palatable neighbours (Tahvanainen and Root, 1972; Atsatt and O’Dowd, 1976; Rausher, 1981). Since the spatial patterns of species are closely related to ecological processes, we believe that quantitative analyses of species spatial patterns can help elucidate the biological characteristics of plant populations (e.g., sexual propagation strategies, seed number, and seed size), intraspecific relationships (competition or facilitation), the relationships of these factors with environmental stresses, and other important ecological processes (Condit et al., 2000; Lin et al., 2011).

However, to our knowledge, previous studies have not empirically assessed the relative importance of individual seed trait spatial patterns under grazing gradients. In this study, we used the dominant alpine grassland species *Kobresia humilis* of the Qinghai–Tibet Plateau as a model plant, starting with the plant functional traits and *K. humilis* individual spatial pattern. We attempted to use current understandings of the cascade effect of seed trait-stem trait-individual spatial patterns to explain the patterns of dominant plant species change along grazing gradients, with the aim of determining the following: (1) seed size, seed number and the relationship of these two values with reproductive stems and vegetative stems along the grazing gradient and (2) spatial pattern changes between reproductive individuals and nonreproductive individuals at varying levels of grazing intensity.

## 2 Materials and methods

### 2.1 Overview of the study area

The experimental grazing area was part of an adaptive management technology platform in the alpine grassland–livestock system in Xihai town, Haibei prefecture, Qinghai Province (36°92’N, 100°93’E), with an average altitude of 3050 m. The area has a plateau continental climate, with an average annual temperature of 1.5°C, with 2,580-2,750 annual sunlight hours, and with an average annual precipitation of approximately 400 mm. The precipitation is mostly concentrated from May to September, with this period accounting for more than 80% of the annual precipitation. The soil type is alpine meadow soil. The total grassland plant community coverage is high (over 85%). In the plant community, *K. humilis* is the most dominant species, and its importance is higher than 30%. The secondary dominant species are *Carex aridula*, *Elymus nutans*, *Poa annua*, *Potentilla acaulis*, etc.

The research subject of this study was *K. humilis*, which is a cold, mesophytic, dense bush, rhizomatous perennial plant that is resistant to cold, drought, radiation, strong wind and barren conditions. Its height ranges from 3-15 cm, with clusters of three blunt ridges. Its leaves are linear and flat and generally shorter than or equal to the height of the stem. The *K. humilis* plants in the study area generally complete heading in mid-May, enter the blooming stage in late May, and begin to form fruit in early June, with fruit maturing in mid-late June. After entering the reproductive period, the reproductive stems of *K. humilis* are persistent and can serve as the main basis for determining whether a plant is a reproductive individual (Dong et al., 2022).

### 2.2 Grazing experimental design

The experimental plots selected in this study before the start of grazing were located in the same continuous grassland, with relatively flat terrain and a relatively uniform environment, which effectively controlled for differences in background and spatial heterogeneity. The grazing experiment began in 2018. The annual grazing time was the alpine grassland plant growth season (June-October), and the grazing period involved grazing at night. The grazing experiment was designed as a randomized block experiment. There were four treatments, including the no grazing treatment (control treatment), light grazing treatment, moderate grazing treatment and heavy grazing treatment. Each grazing treatment had three replicates and a total of 12 experimental plots. Each grazing plot was separated by a 1.2 m high fence. The nongrazing treatment underwent no grazing throughout the year. The area of the light grazing treatment was 0.66 hm^2^, and the carrying capacity was 3.03 Tibetan sheep/hm^2^. The area of the moderate grazing treatment was 0.51 hm^2^, and the carrying capacity was 3.92 Tibetan sheep/hm^2^. The area of the heavy grazing treatment was 0.39 hm^2^, and the carrying capacity was 5.12 Tibetan sheep/hm^2^. According to a pretest performed with no grazing treatment as a reference, the forage utilization rate of the light grazing treatment was 30-35%, the forage utilization rate of the moderate grazing treatment was 50-55%, and the forage utilization rate of the moderate grazing treatment was 65-70% (Liu et al., 2022). There was no supplementary feeding during grazing, and drinking water was added every 2 days. To ensure the consistency of the experiment, one-year-old male Tibetan sheep with a body weight of 30 ± 2 kg were selected as grazing livestock, and their body weights and body conditions were generally the same. To ensure normal livestock feeding and metabolic activities during grazing, the livestock were treated with insecticide before grazing (Yang et al., 2019).

### 2.3 Data acquisition

To obtain relevant data on the reproductive stems of reproductive *K. humilis* individuals in the experimental plot, field sampling was conducted in late June 2021. In each experimental plot, 10 K*. humilis* reproductive individuals with relatively intact organs were randomly selected, for a total of 30 plants in each treatment. The individual plants were collected by cutting with scissors and were immediately brought indoors to shade. A total of 180 plants were sampled in this experiment. To ensure the accuracy of the measured indicators and to minimize errors, an electronic Vernier calliper (0-150 mm) was used in this experiment to measure the lengths of the vegetative stems. After separating the different organs, the vegetative and reproductive stems were counted. The single seed sizes (seed mass, Lönnberg and Eriksson, 2013) of each *K. humilis* plant were measured with an electronic balance (FA2104N), and the seed quantity for each *K. humilis* plant was determined. The fresh plant materials were then placed in an oven at 105°C for 10 min to deactivate the enzymes. The vegetative stems and reproductive stems were dried to constant weight in a blast drying oven at 65°C, and the dry weights of the samples were determined. The weights of the vegetative stems and reproductive stems were measured with a ten-thousandth balance.

A 5 m × 5 m quadrat was randomly established in each grazing treatment and a 50 cm × 50 cm quadrat frame was placed inside, shifting it 100 times from left to right and from top to bottom (the quadrat frame was placed due north and due south), in order to record spatial coordinates for the entire 5 m × 5 m quadrat. We characterized *K. humilis* individuals with reproductive stems as reproductive individuals and individuals without reproductive stems as nonreproductive individuals (Ren et al., 2012). The spatial coordinates of reproductive individuals and nonreproductive individuals were determined, and the number of reproductive individuals and nonreproductive individuals in each sample box was recorded. The relative position of each reproductive individual and nonreproductive individual in the sample square was recorded based on the vertex of the lower left corner of the sample box, and the relative spatial position was represented by the coordinate value (Wiegand and Moloney, 2013).

### 2.4 Data analysis

*K. humilis* is a dense, bushy, herbaceous plant with basal leaves. There is no obvious stem-leaf differentiation. The lower part of the leaf is folded, the upper part is flat, and the width is 1~2 mm. There is no stem–leaf differentiation among the reproductive stems. Therefore, when analysing the resource acquisition ability of *K. humilis*, with its slender plant organs, in the different treatments, we drew on the concept and calculation method of specific leaf area. In this study, in addition to the *K. humilis* phenotypic plant characteristics, resource allocation within the plants, that is, the plant organ unit weight in relation to length, was defined. Therefore, the equation to calculate resource allocation to the reproductive stems and vegetative stems was:

Resource allocation = organ weight/(organ number × organ length)

Regarding the weights, the organ weight is the total weight of the reproductive (vegetative) stems, the number of organs is the number of reproductive (vegetative) stems, and the organ length is the length of the reproductive (vegetative) stems.

One-factor analysis of variance was used to analyse *K. humilis* individual seed number and seed size and resource allocation to reproductive stems and vegetative stems in the no grazing (control), light grazing, moderate grazing and heavy grazing treatments, and Duncan’s multiple comparison was carried out. The data were expressed as the mean ± standard error. The coefficient of variation (coefficient of variation = standard deviation/mean) was used to represent the variation in seed number and seed size. The above statistical analysis was performed in R 3.6.1.

To further clarify the potential relationship between the reproductive traits of reproductive individuals and seed number and seed size under grazing treatments, a structural equation model of seven observed variables (grazing treatment, number of reproductive stems, weight of reproductive stems, number of vegetative stems, weight of vegetative stems, number of seeds and seed size) was constructed.

A trade-off analysis of seed number, seed size and resource allocation to reproductive stems and vegetative stems was conducted. A simple way to quantify the trade-off between the two functions is to calculate the root mean square deviation between the functions (Lu et al., 2014). The dotted line represents no trade-off, and the coordinate system is divided into two parts. The root mean square deviation (RMSD) is the vertical distance from the midpoint of the coordinate to the dotted line. The greater the distance is, the stronger the trade-off, and the smaller the distance is, the stronger the synergy.

First, the seed number, seed size, resource allocation to the reproductive stems and resource allocation to the vegetative stems of the reproductive *K. humilis* individuals were subjected to dimensionless processing. The formula used is as follows, and the processed data range was between 0 and 1:


$$ES_{std}=\frac{ES_{obs}-ES_{\min}}{ES_{\max}-ES_{\min}}$$

ES_obs_ represents the observations, and ES_min_ and ES_max_ represent the minimum and maximum values in the observations, respectively.


$$RMSD=\sqrt{\frac{1}{n-1}\sum_{i=1}^{n}{\left(ES_{i}-\bar{ES}\right)}^{2}}$$

ES_i_ represents the ith observed value after dimensionless processing, $\bar{\text{ES}}$ represents the average, and RMSD is the root mean square deviation.

The point pattern analysis method is based on the point coordinates of individuals in the plot. Each individual of the population is regarded as a point in the two-dimensional space. All individuals constitute a spatial distribution point map. Ripley’s K function method is used to analyse the multiscale spatial distribution pattern and correlation of the population. The calculation formula is as follows:


$$K\left(t\right)=\frac{A}{n^{2}}\sum_{i=1}^{n}\sum_{j=1}^{n}\frac{I_{t}\left(u_{ij}\right)}{W_{ij}}\left(i\;\neq \;j\right)$$

where A is the sample area; u_ij_ is the distance between plant i and plant j; n is the total number of individuals of each species in the plot, that is, the total number of points; and t is the number of values > 0. W_ij_ is the weight, which is the ratio of the perimeter of the circle with u_ij_ as the radius and i as the centre to sample area A, which can eliminate the boundary effect.

Similarly, the association analysis of reproductive and nonreproductive *K. humilis* individuals is actually the point pattern analysis of two types of individuals, and is also known as multivariate point pattern analysis. Applying the Ripley K function to bivariates, K_ab_(t) can be estimated by the following formula:


$$K_{ab}\left(t\right)=\frac{A}{n_{a}n_{b}}\sum_{i=1}^{n}\sum_{j=1}^{n}\frac{I_{t}\left(u_{ij}\right)}{W_{ij}}$$

In the formula, n_a_ is the number of reproductive individuals a, and n_b_ is the number of nonreproductive individuals b. The improved formula is as follows:


$$L_{ab}\left(t\right)=\sqrt{K_{ab}\left(t\right)/\pi -t}$$

In this formula, when L_ab_(t)< 0, it indicates that the interspecific relationship between reproductive and nonreproductive *K. humilis* individuals is negative at the t scale; when L_ab_(t) = 0, it indicates that there is no correlation between reproductive and nonreproductive *K. humilis* individuals on the t scale; and when L_ab_(t) > 0, it indicates that the reproductive and nonreproductive *K. humilis* individuals are positively correlated at the t scale.

The Monte Carlo test was used to fit the trace line to test the significance of the spatial correlation between populations. The spatial association between *K. humilis* reproductive and nonreproductive individuals is indicated by the value of L_ab_(t), with positive associations shown if the value is above the envelope line, no association shown if the value is between the envelope lines, and a negative association shown if the value is below the envelope line.

## 3 Results

### 3.1 Seed size, seed number and the relationship with reproductive individuals

**Figure 1:**
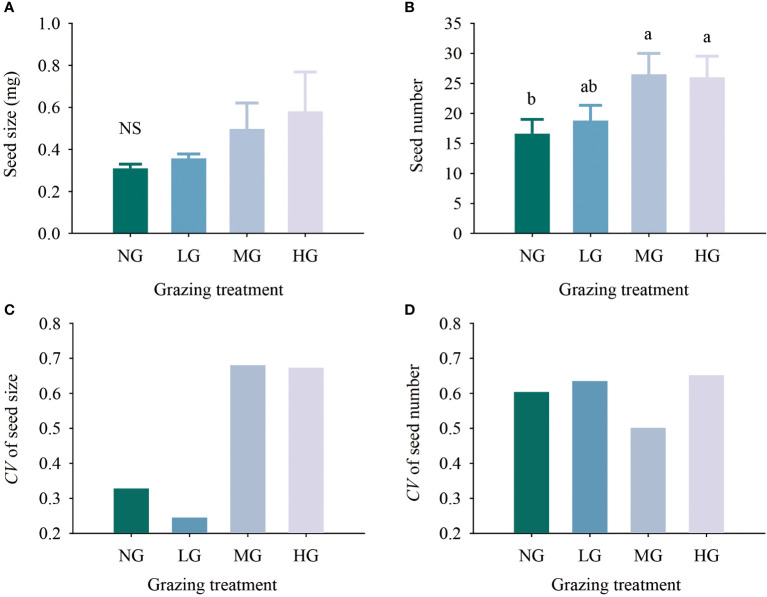
Effects of different grazing treatments on seed size **(A)**, seed number **(B)**, seed size variation coefficient **(C)** and seed number variation coefficient **(D)** of *Kobresia humilis* in alpine grassland (mean ± standard error). NG, no grazing; LG, light grazing; MG, moderate grazing; HG, heavy grazing. Different lowercase letters indicate significant differences between different grazing treatments (*P*< 0.05), and NS indicates no difference between treatments.

The results of the one-factor analysis of variance showed that seed size increased with increasing grazing intensity, but there was no significant difference in seed size among the treatments. The number of seeds under the moderate and heavy grazing treatments was 59.64% and 56.63% higher than that under no grazing (*P*< 0.05). The larger the coefficient of variation in seeds was, the stronger the seed trait plasticity. The coefficients of variation in seed size and seed number were higher in the heavy grazing treatment than in the other treatments, with both coefficients being greater than 0.6. In terms of seed size, the coefficients of variation for the light grazing treatment and nongrazing treatment were less than 0.35, while in terms of seed number, the coefficient of variation for each grazing treatment was greater than 0.5, and the coefficients of variation for the light grazing treatment and nongrazing treatment were greater than 0.6 (**Figure 1**).

**Figure 2:**
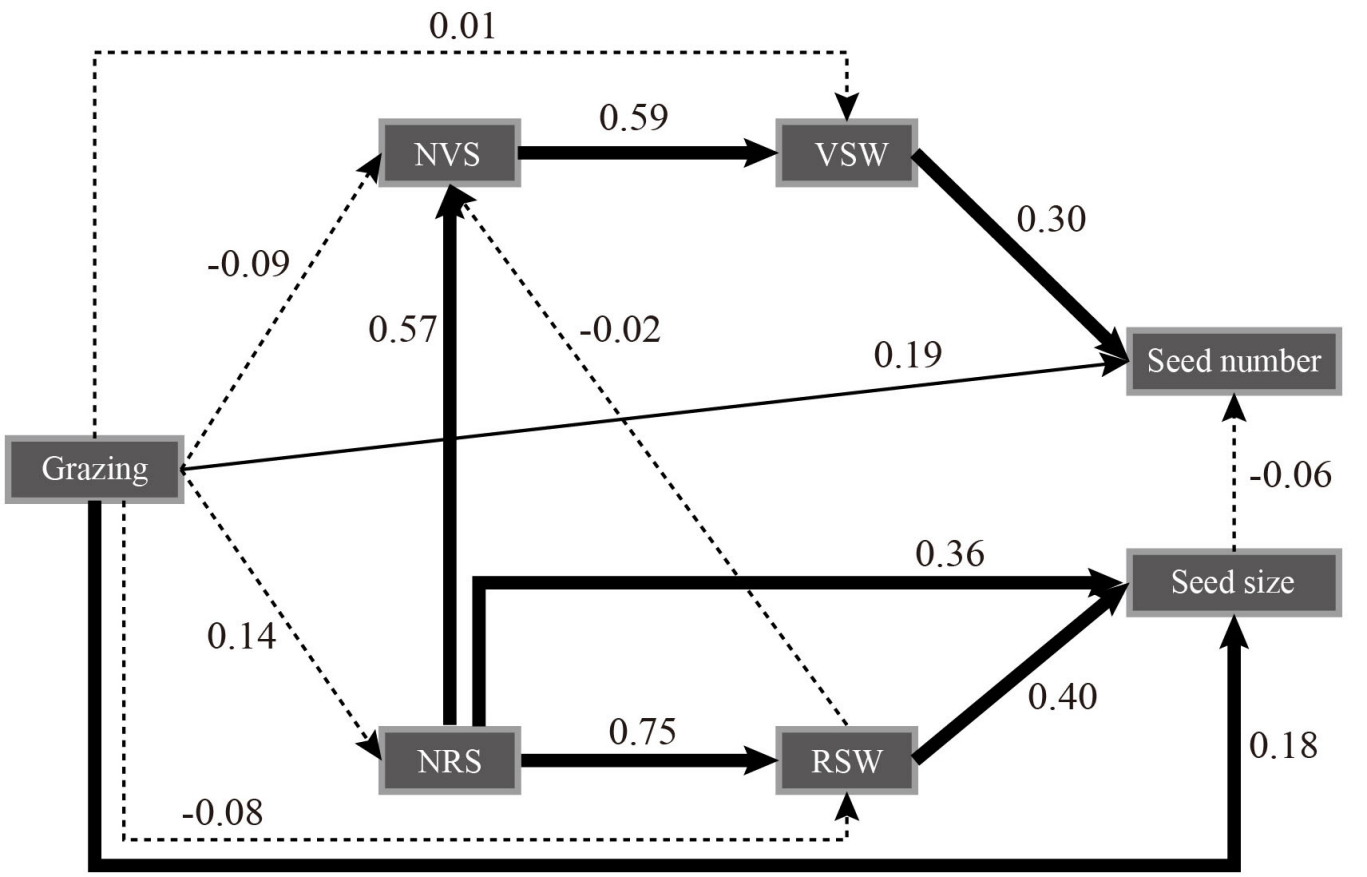
Structural equation model of grazing treatment on seed number and size, number and weight of reproductive stems, and number and weight of vegetative stems of *Kobresia humilis*. Chi-square = 10.407; degrees of freedom = 7; probability level = 0.167. NVS, number of vegetative stems; VSW, vegetative stem weight; NRS, the number of reproductive stems; RSW, reproductive stem weight. The thick solid line indicates *P*< 0.01, the thin solid line indicates *P*< 0.05, and the dotted line indicates *P* > 0.05.

**Figure 3:**
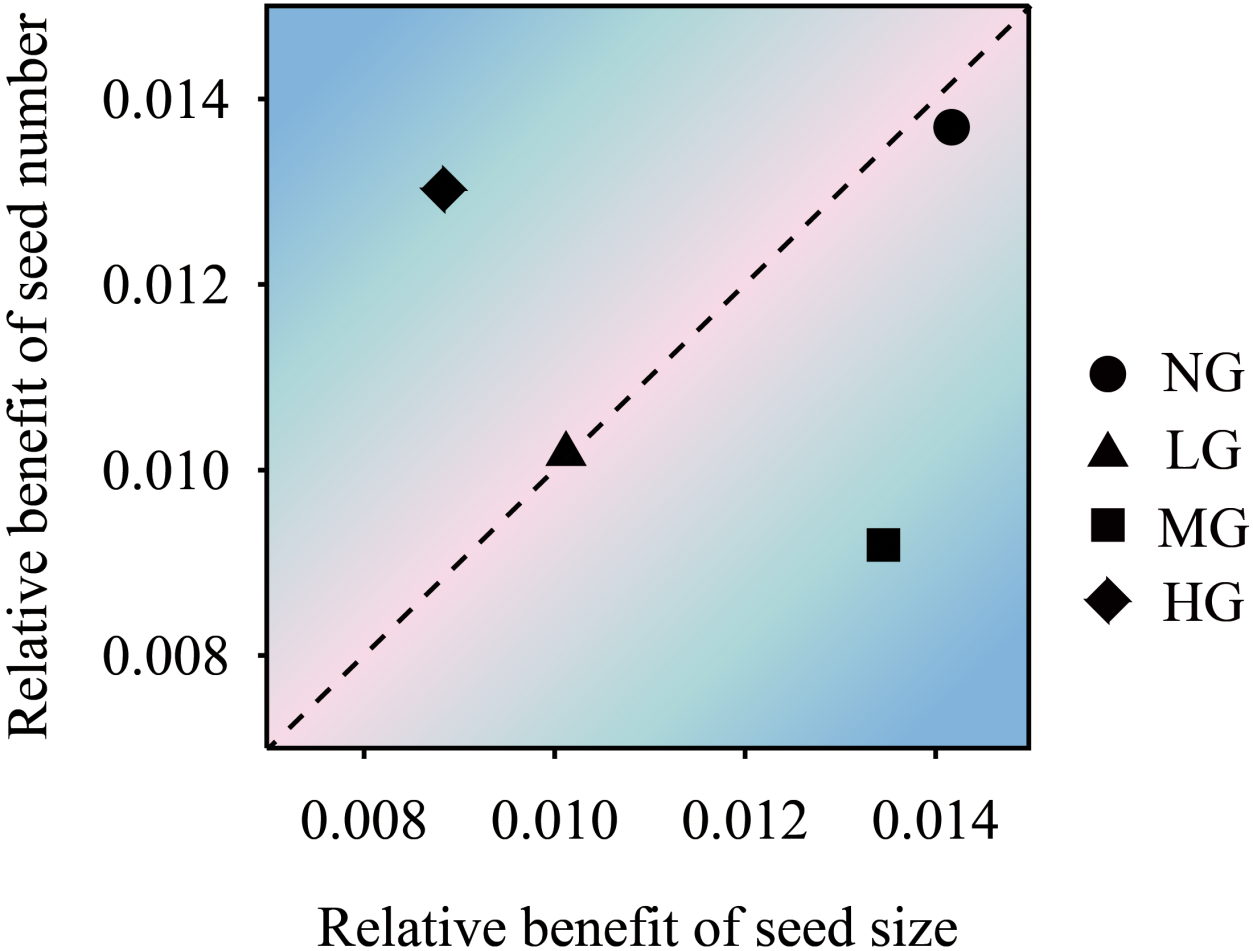
The trade-off relationship between *K. humilis* seed number and seed size under different grazing treatments. NG, no grazing; LG, light grazing; MG, moderate grazing; HG, heavy grazing.

The structural equation model showed that grazing treatment had a significant positive effect on seed number and seed size (*P*< 0.05) and a negative effect on reproductive stem weight but a positive effect on reproductive stem number. The number of reproductive stems and the weight of reproductive stems had a very significant positive effect on seed size. The number of reproductive stems had a significant positive effect on the number of vegetative stems and indirectly affected the number of seeds through the weight of the vegetative stems. Seed size had a negative effect on the number of seeds. Grazing had a negative effect on the number of vegetative stems but a positive effect on the weight of vegetative stems (**Figure 2**). The trade-off analysis of seed size and seed number showed that the moderate grazing treatment and nongrazing treatment affected seed size more than the other variables, and the trade-off value of the moderate grazing treatment was greater than that of the nongrazing treatment. The trade-off in the heavy grazing treatment was related to seed number, and there was almost no trade-off in the light grazing treatment (**Figure 3**).

The above results showed that the *K. humilis* seed size increased with increasing grazing intensity. The medium grazing treatment and heavy grazing treatment significantly increased the number of seeds, and grazing improved the trait plasticity of the seeds. The seed traits had strong plasticity under the heavy grazing treatment.

### 3.2 Resource allocation and trade-off between reproductive stems and vegetative stems

**Figure 4:**
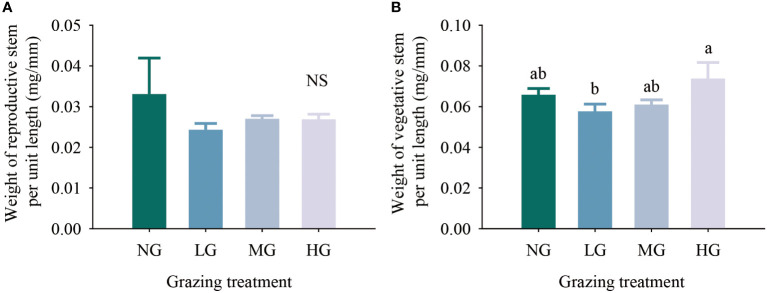
Effects of different grazing treatments on reproductive stem resource input **(A)** and vegetative stem resource input **(B)** of *Kobresia humilis* in alpine grassland (mean ± standard error). NG, no grazing; LG, light grazing; MG, moderate grazing; HG, heavy grazing. Different lowercase letters indicate significant differences between different grazing treatments (*P*< 0.05), and NS indicates no difference between treatments.

**Figure 5:**
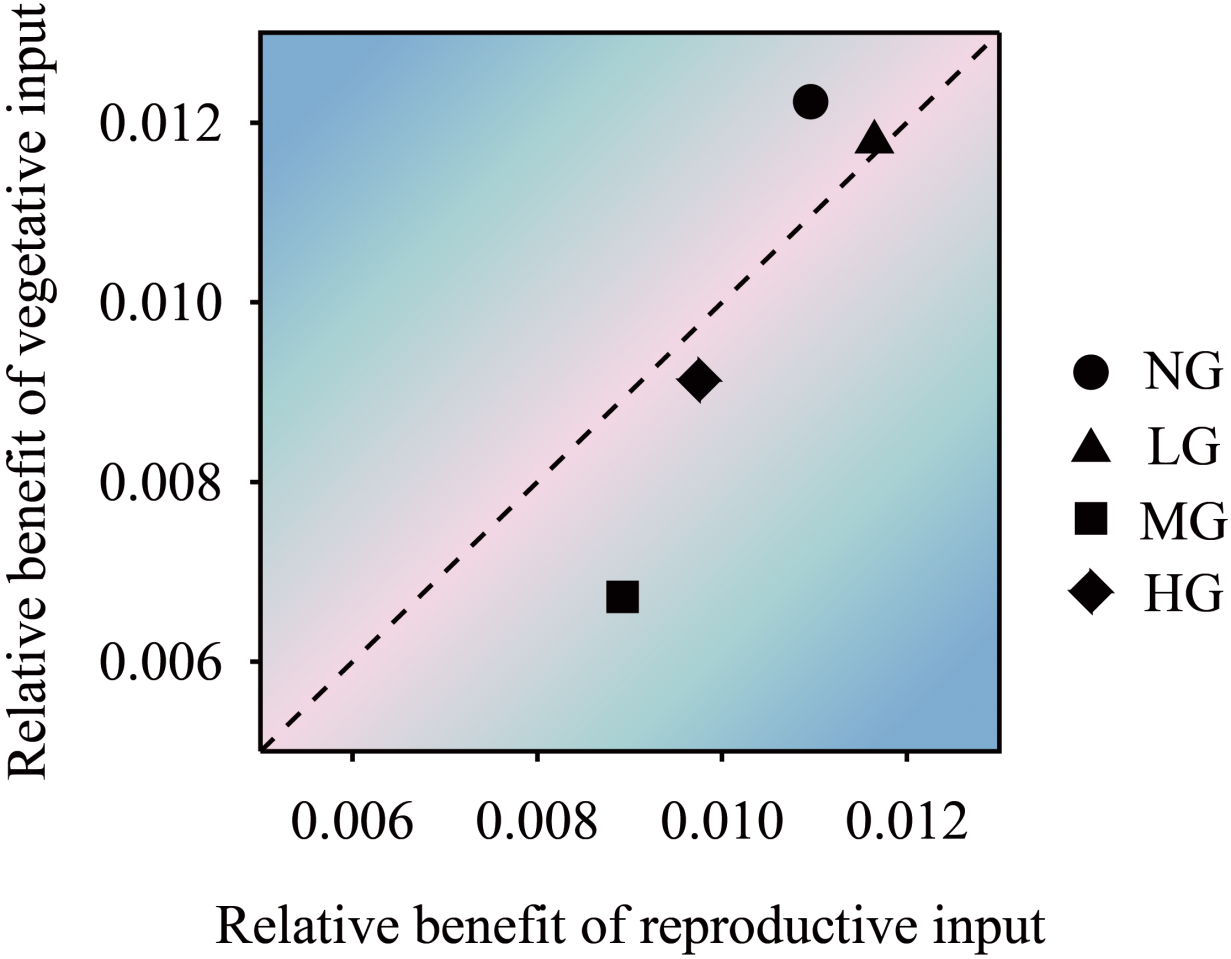
The trade-off relationship between *K. humilis* reproductive and vegetative stem resource inputs of under different grazing treatments. NG, no grazing; LG, light grazing; MG, moderate grazing; HG, heavy grazing.

The results of the one-factor analysis of variance showed that compared with the no grazing treatment, the grazing treatments did not affect resource allocation to the reproductive stems and vegetative stems per unit length of *K. humilis* reproductive individuals. There was no significant difference in the resource allocation to the vegetative stems per unit length among treatments, but the resource allocation to the vegetative stems per unit length was significantly higher in the heavy grazing treatment than in the light grazing treatment (**Figure 4**). The trade-off results showed that the resources allocated to reproductive stems and vegetative stems by reproductive individuals were greater in the moderate grazing treatment than in the no grazing treatment, and both treatments resulted in resource allocation to vegetative stems. The heavy grazing treatment resulted in resource allocation to reproductive stems, and the light grazing treatment had almost no trade-off (**Figure 5**). The above results indicate that under relatively frequent feeding, *K. humilis* is more inclined to allocate resources to reproductive stems in the breeding period.

### 3.3 Weight and pattern of reproductive and nonreproductive individuals

**Figure 6:**
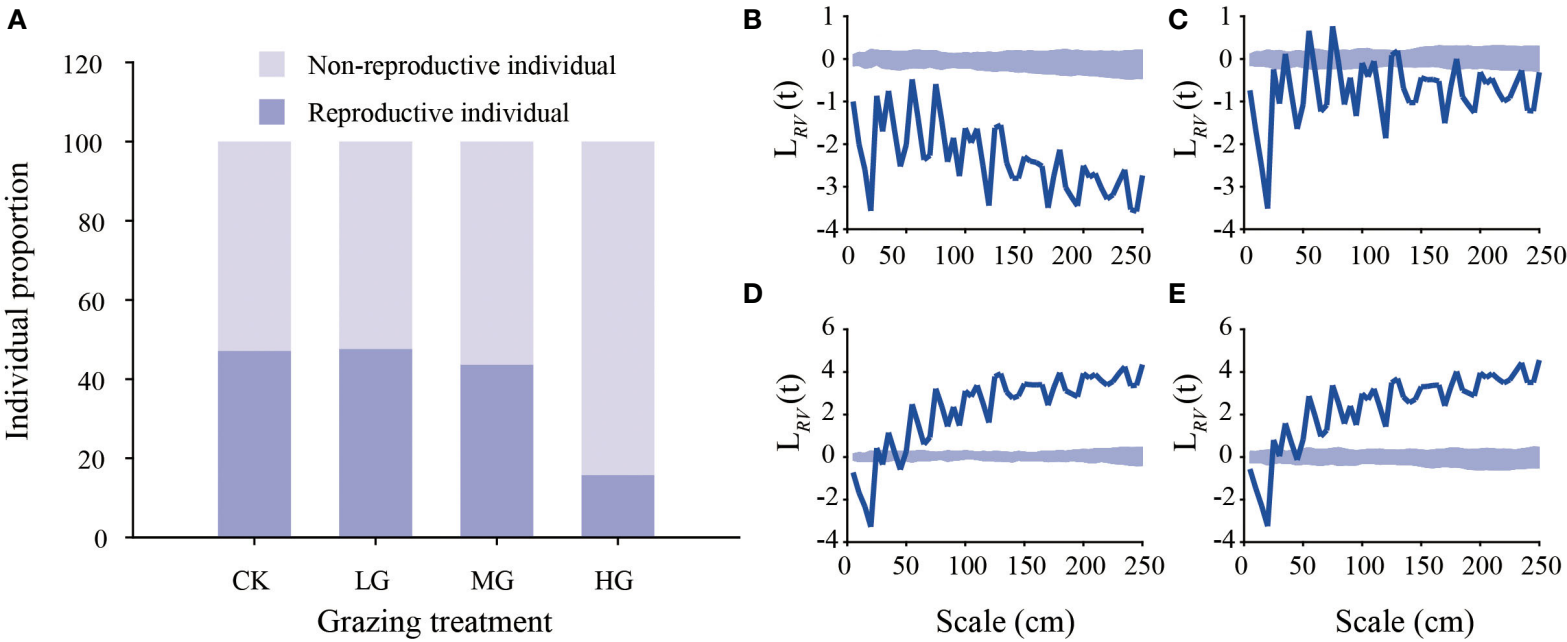
Proportion of reproductive and nonreproductive *Kobresia humilis* individuals under different grazing treatments **(A)** and point pattern analysis of reproductive and nonreproductive *Kobresia humilis* individuals under different grazing treatments. The thick blue line represents the measured value, and the shaded area represents the upper and lower confidence intervals. When the thick blue line is located above the confidence interval, the nonreproductive and reproductive individuals are spatially positively correlated. When the thick blue line is located below the confidence interval, the nonreproductive and reproductive individuals are spatially negatively correlated. No grazing treatment **(B)**, light grazing treatment **(C)**, moderate grazing treatment **(D)**, heavy grazing treatment **(E)**. NG, no grazing; LG, light grazing; MG, moderate grazing; HG, heavy grazing.

The ratio of reproductive individuals (RIs) to nonreproductive individuals (NRIs) showed that the number of reproductive individuals in the heavy grazing treatment decreased by approximately 65.84 compared with that in the no grazing treatment, light grazing treatment and moderate grazing treatment. Within an area of 500 × 500 cm, 3690 (NRIs = 1871, RIs = 1819), 3833 (NRIs = 1985, RIs = 1848), 2116 (NRIs = 986, RIs = 1130) and 2697 (NRIs = 2234, RIs = 463) *K. humilis* individuals were observed in the no grazing, light grazing, moderate grazing and heavy grazing treatments, respectively (**Figure 6A**). The results of the point pattern analysis showed that in the nongrazing treatment, the reproductive and nonreproductive *K. humilis* individuals showed a negative spatial correlation at all scales. In the light grazing treatment, the reproductive and nonreproductive individuals showed a positive spatial association at 55 cm and 75 cm and no association at 35 cm, 80 cm, 110 cm, 125 cm, and 130 cm. In addition to the above scales, both reproductive and nonreproductive individuals were, in spatial terms, negatively correlated. In the moderate grazing treatment and heavy grazing treatment, the distance between reproductive individuals and nonreproductive individuals was less than 20 cm, showing a negative spatial correlation. When this distance was > 50 cm, there was a positive spatial correlation, and the other scales indicated no correlation fluctuation (**Figure 6**). These results indicate that grazing intensity and spatial scale can enhance the spatial aggregation of *K. humilis*.

## 4 Discussion

By studying the seed traits of *K. humilis* and their relationship with reproductive individuals, the relationship between reproductive stems and vegetative stems, and the weight and spatial pattern of reproductive individuals and nonreproductive individuals, we addressed two scientific problems: (1) seed size and number and their relationship with reproductive stems and vegetative stems along a grazing gradient and (2) spatial pattern changes between reproductive individuals and nonreproductive individuals under varying grazing intensities. First, with increasing grazing intensity, the *K. humilis* seed size and number showed an upwards trend, and under heavy grazing, the seeds showed high trait plasticity. Second, the grazing treatments did not affect the resource input of reproductive stems and vegetative stems per unit length of reproductive *K. humilis* individuals, but through trade-off analysis, it was found that in the breeding period *K. humilis* was more inclined to allocate resources to reproductive stems under relatively frequent feeding. Finally, with the increase in grazing intensity, the spatial correlation between reproductive and nonreproductive *K. humilis* individuals gradually changed from a negative correlation to a positive correlation, which increased the *K. humilis* spatial aggregation.

Variation in seed size and seed number is a comprehensive reflection of life history traits and resource regulation strategies, with important ecological and evolutionary significance (Dong et al., 2022). This variation affects population dynamics and interspecific and intraspecific interactions through ecological processes such as distribution limitation, dispersal pathway variation, and regeneration of species populations (Muller-Landau, 2003) and is the core issue of plant fitness research. The results of Xie et al. (2016) showed that in comparison to nongrazing treatments, grazing treatments increase the seed size and seed number of dominant species. This result is consistent with our research results. The seed size increased with increasing grazing intensity, and the moderate and heavy grazing treatments significantly increased the number of *K. humilis* seeds (**Figure 1**). This increase occurred because Cyperaceae species do not grow independently in the studied grassland but are instead mixed with a variety of species, and Tibetan sheep preferentially feed on more palatable gramineous plants and legumes during their feeding process. Thus, to a certain extent, these plants jointly protect *K. humilis*, as this species is a low feeding priority (see, for example, Eskelinen, 2008). This scenario indirectly increased the habitat sites available for rapid *K. humilis* growth and development, allowing the species to occupy more land resources and form a ‘ring’ plant cluster; thus, it was able to obtain more resources and invest them in sexual reproduction, forming a continuous circle. These results support the grazing optimization hypothesis as it relates to seed trait patterns.

In addition to confirming the grazing optimization hypothesis, it is also important to consider the impacts of grazing on seed size and the implications for plant development. Our study showed that the seed size coefficient of variation was higher in the moderate and heavy grazing treatments than in the other treatments (**Figure 1**), indicating that the seed traits under heavy grazing had stronger plasticity or seed polymorphism than those under the other treatments. Research has shown that due to asymmetric competition between seeds of different sizes, large-seed plants often have advantages, such as higher survival rates, faster germination rates, and stronger seedling growth, when in direct competition with small-seed plants (Lönnberg and Eriksson, 2013). Small seeds usually find new unoccupied habitats due to their quantitative advantages (Rees and Westoby, 1997), suggesting that under moderate grazing and heavy grazing, *K. humilis* can produce not only small seeds for long-distance dispersal but also large seeds for improving survival probability in highly competitive habitats. This scenario improves the *K. humilis* competition-colonization strategy, indicating that seed size variation within individual plants is an important mechanism for coping with frequent animal interference and maintaining population stability.

Regarding resource allocation, in this study, grazing did not significantly reduce the resources allocated to the reproductive and vegetative stems of *K. humilis* individuals. This result is in contrast to the hypothesis that grazing negatively affects plants (Zhang et al., 2022). We believe that under heavy grazing and moderate grazing with relatively frequent feeding, the reproductive stem growth of individual *K. humilis* plants during the breeding period regulates the growth of vegetative stems; that is, the trade-off between reproductive stems and vegetative stems favours reproductive stems, which was shown in the trade-off analysis. The structural equation model further verified our hypothesis that grazing had a significant positive effect on the number of reproductive stems, the weight of reproductive stems, the number of seeds and the size of seeds, which was consistent with the results of Liu et al. (2020). Thus, under the environmental conditions of frequent livestock foraging stress over a long time, the trade-off between reproductive stems and vegetative stems is obviously not a wise ecological strategy if it favours the vegetative stems because this would increase the survival cost for *K. humilis* and would not be conducive to population regeneration and survival. In addition, the structural equation model also showed that grazing did not significantly affect the number of reproductive stems, the weight of reproductive stems, the number of vegetative stems, or the weight of vegetative stems. Therefore, we firmly believe that reproductive individuals allocate resources in an efficiently controlled manner. In addition, during the long-term interaction between plants and large livestock, grazing can activate and change the resource allocation pattern of dominant species in grasslands.

Both competition and facilitation play an important role in shaping the spatial structure of plant populations (Getzin et al., 2008; Erfanifard et al., 2018; Liu et al., 2020). Our results showed that with the increase in grazing intensity, the spatial pattern of *K. humilis* reproductive and nonreproductive individuals changed from a negative association at a full scale in the no grazing treatment to a small-scale negative correlation and a large-scale positive correlation in the moderate and heavy grazing treatments. This result is similar to the results reported by Lv et al. (2019) and Liu et al. (2020); that is, grazing intensity enhances the spatial aggregation of dominant species in grasslands. In this study, this scenario may have occurred because in the nongrazing treatment, *K. humilis* survival and growth were mainly affected by competition between individuals; that is, different individuals were able to infringe upon each other’s living space and resources. In the moderate grazing treatment, where plants were disturbed by Tibetan sheep feeding, grazing altered the colonization and extinction of palatable species (such as Poaceae species), resulting in the exclusion of competitively inferior species (such as Fabaceae species). Thus, as a result of grazing, grasslands have considerable spare niches, which increase the availability of sites for seed bank plants and increase the *K. humilis* reproduction and distribution range (Olff and Ritchie, 1998), resulting in a positive association between reproductive and nonreproductive individuals at a larger scale and enhancing the mutual benefits and resource availability between individuals (Bertness and Callaway, 1994). At a smaller scale, studies suggest that plant distribution patterns are mainly affected by distribution constraints and seedling recruitment processes (Shen et al., 2013). However, our study showed that frequent animal disturbance induced more and larger polymorphic *K. humilis* seeds. Obviously, even under heavy grazing, *K. humilis* populations were not limited by distribution. In contrast, abundant and polymorphic seeds will gather around reproductive individuals, and the free niche released by heavy grazing will cause intraspecific competition between seedlings and reproductive individuals. Aggregation of mature reproductive individuals with large numbers of seedlings at a small scale also seems to be significant because: 1) Livestock tend to feed on more nutritious reproductive organs than on nonreproductive organs. The repellent-plant hypothesis states that a target individual will not be fed on by animals when forming a neighbour relationship with a plant with lower nutritional value than itself. Over time, livestock will stay away from low-quality plants or patches, and reproductive individuals will avoid being fed upon. 2) Because the probability of *K. humilis* individuals being damaged by feeding is 0-1, the more seedlings the older plants produce, the more likely it is that reproductive *K. humilis* individuals will adopt this risk-sharing strategy to reduce the risk of individual feeding. This scenario suggests that in the moderate or even heavy grazing treatments, the change in the intraspecific relationship from a negative association to a positive association with the increase in distance between reproductive and nonreproductive individuals was an ecological strategy for population survival.

In summary, in this study, grazing was conducive to *K. humilis* sexual reproduction, to increasing the *K. humilis* seed size and number and to promoting the *K. humilis* acquisition of resources and the investment of resources in sexual reproduction. Moreover, grazing intensity enhanced the *K. humilis* spatial aggregation, resulting in the development of an ecological strategy conducive to the survival of the *K. humilis* population. The beneficial effect of grazing on the development of this dominant species indicated that moderate grazing intensity was conducive to maintaining the stability of the ecosystem and was the key to achieving sustainable development of grassland animal husbandry.

## 5 Conclusion

Our study showed that grazing could activate and change the resource allocation pattern of a dominant species in a grassland and that it had significant positive effects on the number of reproductive stems, the weight of reproductive stems, the number of seeds and the size of seeds. Seed size variation within plant individuals is an important mechanism for coping with frequent animal interference and maintaining population stability. Under a grazing gradient, the change in the intraspecific relationship from a negative association to a positive association with the increase in distance between reproductive and nonreproductive individuals is an ecological strategy for population survival.

## Data availability statement

The raw data supporting the conclusions of this article will be made available by the authors, without undue reservation.

## Author contributions

ZQ-Y, WF-C, FB, LY-Z, Experimentation. DQ-M, YX-X, YY, ZC-P, CQ, LW-T, Supervision and Research Design. ZQ-Y, WF-C, Review and Drafting. LY-Z, FB, YX-X, YY, Validation and Statistical Analysis. DQ-M, ZC-P, CQ, LWT-, Drafting and Validation. All authors contributed to the article and approved the submitted version.
