# Supplementary material for: Cascading effects of seed-stem-individual spatial patterns along a grazing gradient
Source: Front Plant Sci. 2023 Mar 15;14:1137726. doi: 10.3389/fpls.2023.1137726 (PMC10050678; doi:10.3389/fpls.2023.1137726)
Supplement: Supplementary file 1 [file DataSheet_1.docx]

Supplementary Material

Cascade effect of seed - branch - individual spatial patterns under a grazing gradient

**ZHOU Qin-Yuan1, DONG Quan-Min1,2, Wang Fang-Cao1, LIU Yu-Zhen1, FENG Bin1, YANG Xiao-Xia1, YU Yang1, ZHANG Chun-Ping1, CAO Quan1, LIU Wen-ting1* Correspondence:** LIU Wen-ting, email qhdxlwt@163.com

## Supplementary Figures


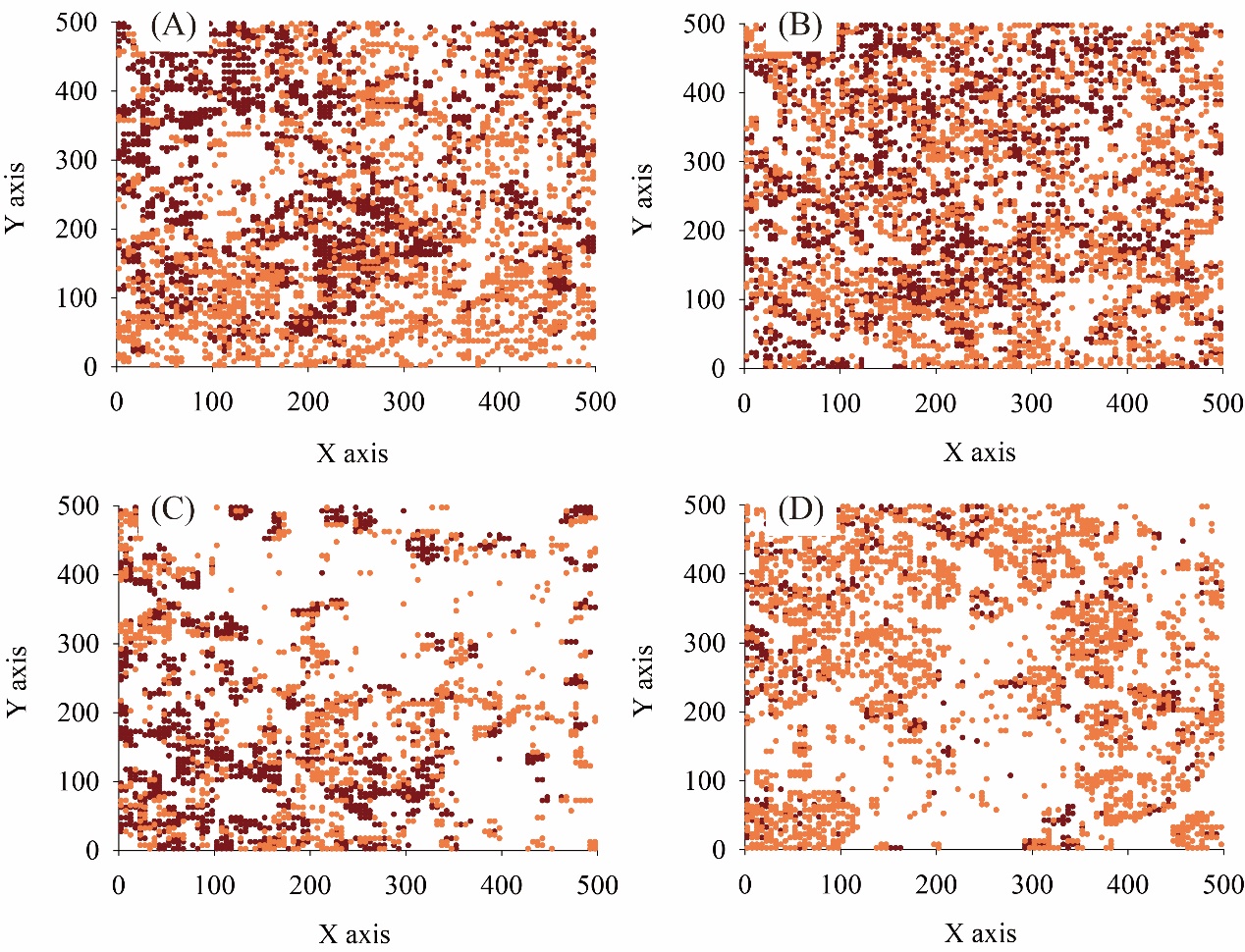


Supplementary Figure 1. Spatial distribution map of reproductive and nonreproductive individuals of *Kobresia pygmaea*
